# The *unfulfilled *gene is required for the development of mushroom body neuropil in *Drosophila*

**DOI:** 10.1186/1749-8104-5-4

**Published:** 2010-02-01

**Authors:** Karen E Bates, Carl S Sung, Steven Robinow

**Affiliations:** 1 Department of Zoology, University of Hawaii, Honolulu, HI 96822, USA

## Abstract

**Background:**

The mushroom bodies (MBs) of *Drosophila *are required for complex behaviors and consist of three types of neurons, γ, α'/β' and α/β. Previously, roles for transcription factors in MB neuronal differentiation have only been described for a subset of MB neurons. We are investigating the roles of *unfulfilled *(*unf*; *HR51*, CG16801) in MB development. *unf *encodes a nuclear receptor that is orthologous to the nuclear receptors fasciculation of axons defective 1 (FAX-1) of the nematode and photoreceptor specific nuclear receptor (PNR) of mammals. Based on our previous observations that *unf *transcripts accumulate in MB neurons at all developmental stages and the presence of axon pathfinding defects in *fax-1 *mutants, we hypothesized that *unf *regulates MB axon growth and pathfinding.

**Results:**

We show that *unf *mutants exhibit a range of highly penetrant axon stalling phenotypes affecting all neurons of the larval and adult MBs. Phenotypic analysis of *unf*^*X1 *^mutants revealed that α'/β' and α/β neurons initially project axons but stall prior to the formation of medial or dorsal MB lobes. *unf*^*Z0001 *^mutants form medial lobes, although these axons fail to branch, which results in a failure to form the α or α' dorsal lobes. In either mutant background, γ neurons fail to develop larval-specific dorsal projections. These mutant γ neurons undergo normal pruning, but fail to re-extend axons medially during pupal development. *unf*^*RNAi *^animals displayed phenotypes similar to those seen in *unf*^*Z0001 *^mutants. Unique asymmetrical phenotypes were observed in *unf*^*X1*^/*unf*^*Z0001 *^compound heterozygotes. Expression of *UAS-unf *transgenes in MB neurons rescues the larval and adult *unf *mutant phenotypes.

**Conclusions:**

These data support the hypothesis that *unf *plays a common role in the development of all types of MB neurons. Our data indicate that *unf *is necessary for MB axon extension and branching and that the formation of dorsal collaterals is more sensitive to the loss of *unf *function than medial projections. The asymmetrical phenotypes observed in compound heterozygotes support the hypothesis that the earliest MB axons may serve as pioneers for the later-born MB neurons, providing evidence for pioneer MB axon guidance in post-embryonic development.

## Background

**Figure 1:**
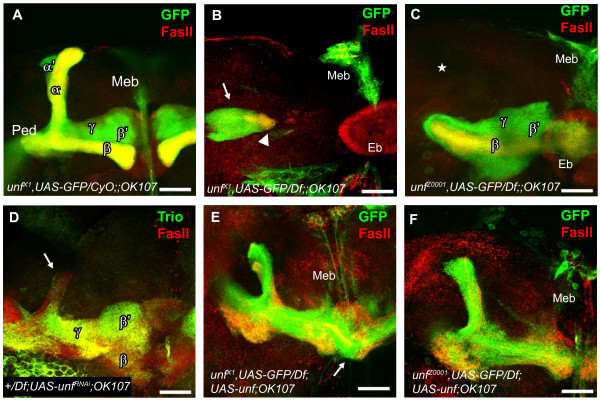
***unf *is required for mushroom body lobe formation**. In the adult brain, the mushroom body (MB) is a paired neuropil structure that comprises five axonal lobes, γ, α'/β', and α/β. Each neuron projects dendrites that contribute to a large dendritic field (calyx), and an axon that travels anteroventrally. MB axons fasciculate with other MB axons forming a peduncle (Ped) before projecting axons medially and dorsally. α' and α axons project dorsally, whereas γ, β', and β axons project medially, forming five distinctive lobes. To visualize the MB lobes, *OK107-GAL4 *was used to drive expression of the *UAS-mCD8GFP *transgene in all MB neurons (Kenyon cells) and their axons. Lobes were distinguished by using anti-Fasciclin II (anti-Fas II) to label α and β lobes and anti-Trio to label α', β', and γ lobes. **(A) **In adult *UAS-mCD8GFP;;OK107-GAL4 *control animals labeled with anti-Fas II (red), all MB lobes have formed. **(B) **In *unf*^*X1 *^mutants, MB axons have formed a peduncle (arrowhead), but have spread out and stalled prior to lobe formation (arrow). **(C) **In *unf*^*Z0001 *^mutants, γ, β', and β axons projected medially, but were disorganized. No dorsal lobes were formed (star). **(D) **This +/*Df2426;UAS-unf*^*RNAi*^;*OK107-GAL4 *adult displayed dramatically reduced dorsal lobes in one brain hemisphere (arrow). **(E, F) **In *unf*^*X1 *^and *unf*^*Z0001 *^rescue animals, in which a wild-type *unf *trangene was expressed in all MB neurons in an otherwise mutant background, all MB lobes were present. It is interesting to note that in rescued flies, MB lobes may have fewer axons, and that some medially projecting axons have extended past the midline (E, arrow). Eb, ellipsoid body; Meb, median bundle; Ped, peduncle. Scale bars = 10 μm.

The mushroom bodies (MBs) of *Drosophila melanogaster*, which are required for olfactory learning and other complex behaviors [1,2], are ideal for studying the transcriptional regulation of interneuronal development because they form discrete axonal projections that are well-characterized [3-5] and easily visualized [4,6-9]. Four neuroblasts in each brain hemisphere sequentially generate three types of Kenyon cells, the γ, α'/β', and α/β MB neurons that begin dividing during embryogenesis and continue to divide through development [10,11]. Each neuron projects dendrites that contribute to a large dendritic field in the calyx, and an axon that travels anteroventrally, forming a tightly bundled peduncle before branching medially to form the γ, β', and β lobes, and dorsally to form the α' and α lobes (Figure 1A). The earliest born γ neurons initially extend axons both medially and dorsally during late embryonic and early larval stages. These larval-specific γ axons are then pruned back to the peduncle by 18 hours after puparium formation (APF) and re-extend medially during pupal remodeling; the late larval-born α'/β' and pupal-born α/β neurons do not remodel their axonal projection patterns during metamorphosis [3-5].

Since the three different classes of MB neurons are born sequentially, generate a single dendritic field, project axons that fasciculate prior to branching medially and/or dorsally to form type-specific lobes, it is interesting to consider whether any differentiative events of the γ, α'/β', and α/β neurons are regulated by a common set of genes or whether they utilize independent transcriptional networks. Existing data on the role of transcription factors in MB differentiation provide little insight into this question. The genes *eyeless *[12-14], *tramtrak *[15], *mushroom body miniature *[16,17], *chinmo *[18], *polyhomeotic *[19], and *tailless *[20] regulate proliferation, specification, and viability of MB neurons, events that precede differentiation. *dachshund *(*dac*), *ecdysone receptor B1 *(*EcR-B1*), *ultraspiracle *(*usp*), and *dSmad2 *act in subtype-specific pathways [12,13,21-23], consistent with the hypothesis that the differentiation of the γ, α'/β', and α/β neurons utilize independent transcriptional pathways. *dac *mutants display axonal branching and pathfinding defects in subsets of α'/β' and α/β MB neurons [12,21]. EcR-B1 and its heterodimeric partner, Ultraspiracle (USP), both members of the nuclear receptor superfamily, form an ecdysone-regulated transcription factor that is required for the pruning of MB γ neurons at the outset of metamorphosis [22]. *dSmad2 *regulates the transcription of *EcR-B1 *in MB γ neurons during neuronal remodeling [24]. Thus, whether any differentiative events of the γ, α'/β', and α/β neurons are regulated by a common set of genes has not been previously reported.

In this study we show that the gene *unfulfilled *is required for the development of all three types of MB neurons, supporting the hypothesis that some differentiative events of the three types of MB neurons are regulated by a common set of genes. The *unfulfilled *gene (*unf*; *HR51*, CG16801) encodes the *Drosophila *NR2E3 member of the nuclear receptor superfamily [25]. UNF, like all classical nuclear receptors, contains an amino-terminal transactivational domain, a DNA-binding domain, a hinge region, and a carboxy-terminal ligand-binding domain [26]. *unf *is an ortholog of the *Caenorhabditis elegans *gene *fasciculation of axons defective *(*fax-1*) and the human gene *photoreceptor specific nuclear receptor *(*PNR*) [27]. Both *fax-1 *and *PNR *mutations disrupt developmental events in a limited number of neurons and result in behavioral or sensory deficits. *fax-1 *mutants are uncoordinated and display axon pathfinding and neurotransmitter defects [28-30]. The observed axon pathfinding defects are inferred to be due to the misregulation of *fax-1 *target genes. PNR impacts neuronal identity of vertebrate photoreceptors, functions as a dimer, and acts as a dual function transcriptional regulator, able to act as a transcriptional activator and a transcriptional repressor [31-37].

Based on our previous observations that robust levels of *unf *transcripts accumulate in MB neurons at all developmental stages [25] and the axon pathfinding defects of *fax-1 *mutants [28-30], we hypothesized that *unf *regulates MB axon growth and pathfinding. Phenotypic analysis of *unf *mutants revealed that MB axons stall prior to the formation of the lobes with the exception of the larval-specific γ neurons, which project axons medially, but fail to project dorsally. These axons are pruned appropriately but fail to re-extend during pupal stages. Expression of an *unf *transgene in the MBs in a mutant background rescued the *unf *mutant phenotypes, demonstrating that MB defects of *unf *mutants are due to loss of *unf *function in the MB neurons. These data demonstrate that *unf *is required for the proper formation of γ, α'/β', and α/β lobes, consistent with the hypothesis that at least some differentiative events of the γ, α'/β', and α/β neurons are regulated by a common set of genes.

## Results

### *unf *mutants show a reduction or complete loss of mushroom body lobes

**Figure 2:**
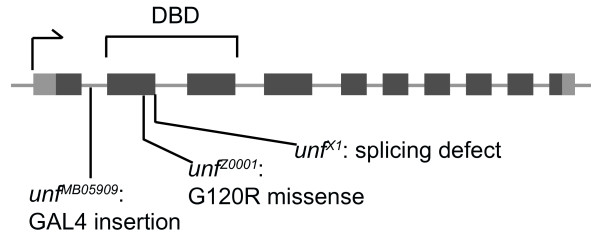
**Summary of *unf *alleles**. The *unf*^*X1 *^allele disrupts the 5' donor splice site of intron 2, whereas the *unf*^*Z0001 *^allele has a missense mutation due to a guanine to adenine transition at base 312 of exon 2, resulting in a glycine to arginine substitution (G120R) [25]. The *unf*^*MB05909 *^line contains a GAL4 insertion in intron 1 of the *unf *gene (FlyBase). DBD, DNA-binding domain.

To test the hypothesis that *unf *regulates MB neuron development, flies of various mutant genotypes (*unf*^*X1*^/*Df2426*, *unf*^*X1*^/*unf*^*X1*^, *unf*^*Z0001*^/*Df2426*, *unf*^*Z0001*^/*unf*^*Z0001*^, *unf*^*X1*^/*unf*^*Z0001*^, *unf*^*MB05909*^/*Df2426*, +/*Df2426*;*UAS-unf*^*RNAi*^;*OK107-GAL4*) were analyzed for aberrant MB phenotypes. All MB axons were visualized by expressing the *UAS-mCD8GFP *(*UAS-GFP*) reporter [9] using the *OK107-GAL4 *transgene, which expresses GAL4-driven green fluorescent protein (GFP) in all MB neurons [38]. Specific lobes were unambiguously identified immunohistochemically using anti-Fasciclin II (anti-Fas II) to label the α/β lobes [6] or anti-Trio to label the α'/β' lobes; both antibodies weakly label the γ lobes [39]. The *unf*^*X1 *^and *unf*^*Z0001*^alleles have been characterized previously [25], while the *unf*^*MB05909 *^allele has been recently identified (FlyBase; Figure 2). All five MB lobes were present and morphologically normal in adult control animals: α and α' lobes projected dorsally, whereas γ, β, and β' axons projected medially stopping at the median bundle (Figure 1A). MB axons of adult *unf*^*X1*^/*Df2426 *hemizygous (*unf*^*X1*^, *UAS-GFP/Df2426;;OK107-GAL4*; Figure 1B) and *unf*^*X1*^/*unf*^*X1 *^homozygous (*unf*^*X1*^/*unf*^*X1*^, *UAS-GFP;;OK107-GAL4*) mutants labeled with anti-Fas II projected anteroventrally, forming a peduncle, but stalled prior to the formation of discrete lobes. Axons that reached the heel region of the MB tended to spread out, but did not extend axons medially or dorsally (Figure 1B; compare Additional file 1 to Additional files 2 and 3). *unf*^*X1*^/*Df2426 *hemizygotes labeled with anti-Trio rather than anti-Fas II confirmed that γ and α'/β' *unf *mutant MB neurons initially projected axons forming a peduncle, but that these axons stalled prior to the formation of lobes (data not shown). MB lobes were never observed in *unf*^*X1*^/*Df2426 *hemizygotes or *unf*^*X1*^/*unf*^*X1 *^homozygotes (Table 1, rows 8 and 9). In contrast, all MB lobes were observed in all control genotypes (*w*^*1118*^, *unf*^*X1*^/+, *Df2426/CyOGFP*, *UAS-GFP;;OK107-GAL4 *controls, and *unf*^*X1*^, *UAS-GFP/CyOGFP;;OK107-GAL4 *control siblings; Table 1, rows 1, 2, 4, 5, and 6). We did not observe a reduced number of MB neurons in *unf *mutants (compare Additional file 1 to Additional files 2 to 5).

**Table 1:** Mushroom body phenotypes in *unf *mutants and rescue animals.

Row	Genotype	All lobes present adult/larvae (%)	All lobes missing adult/larvae (%)	Dorsal lobes missing adult/larvae (%)	Missing a dorsal or medial lobe adult/larvae (%)	*n *adult/larvae
	**Controls**					
1	*w*^*1118*^	100	0	0	0	10
2	*unf*^*X1*^/+	100/100	0/0	0/0	0/0	12/8
3	*unf*^*Z0001*^/+	100/100	0/0	0/0	0/0	7/15
4	*Df/CyOGFP*	100/100	0/0	0/0	0/0	8/8
5	*UAS-GFP;;OK107*	100	0	0	0	10
6	*unf*^*X1*^, *UAS-GFP/CyOGFP;;OK107*	100	0	0	0	15
7	*unf*^*Z0001*^, *UAS-GFP/CyOGFP;;OK107*	90	0	10	0	10
						
	***unf *mutants and gene knock down**					
8	*unf*^*X1*^,*UAS-GFP/Df;;OK107*	0/0	100*/0	0/90*	0/10	15/11
9	*unf*^*X1*^,*UAS-GFP/unf*^*X1*^;;*OK107*	0	100*	0	0	7
10	*unf*^*Z0001*^/*Df, UAS-GFP;;OK107*	0/0	0/0	100*/100*	0/0	13/6
11	*unf*^*Z0001*^/*unf*^*Z0001*^,*UAS-GFP;;OK107*	37.5	0	37.5	25	8
12	*unf*^*MB05909*^/*Df, UAS-GFP;;OK107*	100	0	0	0	14
13	+/*Df;UAS-unf*^*RNAi*^;*OK107*	50	0	40	10	10
14	*unf*^*X1*^,*UAS-GFP/unf*^*Z0001*^;;*OK107*	0	13	47	40	15
						
	***unf *transgenic rescues**					
15_a_	*unf*^*X1*^,*UAS-GFP/Df;UAS-unf*^*rsqF*^/+;*OK107*	100*/83*	0/0	0/0	0/17	11/6
16_b_	*unf*^*Z0001*^/*Df, UAS-GFP/UAS-unf*^*rsqF*^/+;*OK107*	100*	0	0	0	8
17_c_	*unf*^*X1*^,*UAS-GFP/Df;UAS-unf*^*rsqC*^/+;*OK107*	86*	0	0	14	7
						
	***unf *rescue controls**					
18_a_	*unf*^*X1*^,*UAS-GFP/Df;TM3Sb/*+;*OK107*	0	100	0	0	6
19_a_	*unf*^*X1*^,*UAS-GFP/CyO;UAS-unf*^*rsqF*^/+;*OK107*	100	0	0	0	9
20_a_	*unf*^*X1*^,*UAS-GFP/CyO;TM3Sb/*+;*OK107*	100	0	0	0	4
21_a_	*Df/CyOGFP;UAS-unf*^*rsqF*^/+;*OK107*	100	0	0	0	5
22_a_	*Df/CyOGFP;TM3Sb*/+;*OK107*	100	0	0	0	7
23_b_	*unf*^*Z0001*^/*Df, UAS-GFP;TM3Sb*/+;*OK107*	0	0	100	0	7
24_c_	*unf*^*X1*^,*UAS-GFP/Df;TM3Sb/*+;*OK107*	0	100	0	0	9
25	*unf*^*X1*^,*UAS-GFP/Df;UAS-lacZ/*+;*OK107*	0	100	0	0	9
26	*UAS-GFP;UAS-unf*^*rsqF*^/+;*OK107*	100	0	0	0	5

Data are presented as percentages of whole brains that exhibit the phenotype. Control and mutant animals are 0- to 2-day adults, whereas rescue and rescue control animals are 72- to 96-hour pupae. All larvae are third instars. Single entries are adult or late pupae. Rescues and the corresponding control siblings are noted by matching subscript letters in rows. Asterisks indicate *P*-values of < 0.01 from the Fisher exact test. Abbreviations: *Df*, *Df(2R)ED2426*; *UAS-GFP*, *UAS-mCD8GFP*; *OK107*, *OK107-GAL4*.

*unf*^*Z0001*^/*Df2426 *hemizygous (*unf*^*Z0001*^,*UAS-GFP/Df2426;;OK107-GAL4*; Figure 1C) adults formed only medial lobes and displayed γ axons that splayed as they approached the midline compared to the compact bulb-like organization of γ lobes in wild-type animals (Figure 1C; Table 1, row 10; Additional files 4 and 5). α'/β' and α/β neurons of *unf*^*Z0001*^/*Df2426 *hemizygotes projected axons medially, but rarely projected dorsal collateral axons (data not shown). Dorsal lobes were observed more frequently in *unf*^*Z0001*^/*unf*^*Z0001 *^homozygotes (*unf*^*Z0001*^/*unf*^Z0001^,*UAS-GFP/Df2426;;OK107-GAL4*; Table 1, row 11). The observation that dorsal lobes were observed more frequently in *unf*^*Z0001*^/*unf*^*Z0001 *^homozygotes than in *unf*^*Z0001*^/*Df2426 *hemizygotes supports the hypothesis that the *unf*^*Z0001 *^allele is a hypomorph [25]. All MB lobes were observed in control genotypes (*w*^*1118*^, *unf*^*Z0001*^/+, *Df2426/CyOGFP*, *UAS-GFP;;OK107-GAL4 *controls); however, missing dorsal lobes were observed in 1 of 10 *unf*^*Z0001*^,*UAS-GFP/CyOGFP;;OK107-GAL4 *control siblings (Table 1, rows 1, 3, 4, 5, and 7). *unf*^*MB05909*^/*Df2426 *hemizygotes (*unf*^*MB05909*^/*Df2426,UAS-GFP;;OK107-GAL4*) displayed all MB lobes and did not display any abnormal MB phenotypes (Table 1, row 12), suggesting that the GAL4 insertion in the *unf*^*MB05909 *^line does not disrupt *unf *gene function.

To independently test whether *unf *plays a role in MB neuron development, a *UAS-unf*^*RNAi *^line was crossed to the *OK107-GAL4 *line to generate animals in which *unf *levels were reduced in the MBs via RNA interference (RNAi). Adult brains were double-labeled with anti-Fas II and anti-Trio to visualize the five MB lobes. When *OK107-GAL4 *was used to drive *UAS-unf*^*RNAi *^in a wild-type background, normal MBs were observed (data not shown). However, when *OK107-GAL4 *was used to drive *UAS-unf*^*RNAi *^in *unf*^+^/*Df2426 *hemizygotes (*unf*^+^/*Df2426;UAS-unf*^*RNAi*^;*OK107-GAL4*) 50% of brains displayed dramatically reduced dorsal lobes or were missing dorsal lobes bilaterally or unilaterally (Figure 1D; Table 1, row 13). These RNAi data are consistent with the analyses of *unf*^*X1 *^and *unf*^*Z0001 *^mutants, supporting the hypothesis that *unf *is necessary for axon extension and branching in all MB neurons and that dorsal collaterals are more sensitive to loss of *unf *function than medial projections.

### *unf *expression in the mushroom bodies rescues lobe formation

We tested whether expression of *unf *in the MBs was sufficient to rescue the phenotypes observed in *unf *mutants by driving expression of a *UAS-unf *transgene with the *OK107-GAL4 *transgene. Interestingly, all *UAS-unf*^*rsqF*^;*OK107-GAL4 *flies developed to late pupal stages, but failed to eclose. The failure to eclose is probably due to *OK107-GAL4*-driven expression of *unf *in regions other than the MBs causing a disruption that prevents further development. We therefore assessed the MBs of rescued animals at late pupal stages, 72 to 96 hours APF. At this late developmental time in wild-type pupae, the MBs are indistinguishable from those of adult MBs [4]. Medial and dorsal MB lobes were observed in all *unfX1/Df2426 *rescued (*unf*^*X1*^,*UAS-GFP/Df2426;UAS-unf*^*rsqF*^/+;*OK107-GAL4*; Figure 1E) and *unf*^*Z0001*^/*Df2426 *(*unf*^*Z0001*^,*UAS-GFP/Df2426;UAS-unf*^*rsqF*^/+;*OK107-GAL4*; Figure 1F) rescued pupae. In contrast, MB lobes were not observed in *unf*^*X1*^/*Df2426 *control siblings (*unf*^*X1*^,*UAS-GFP/Df2426;TM3*/+;*OK107-GAL4*) that lacked the *UAS-unf*^*rsqF *^transgene (Table 1, compare rows 15 and 18). Similarly, only medial lobes were observed in *unf*^*Z0001*^/*Df2426 *control siblings (*unf*^*Z0001*^*/Df2426,UAS-GFP;TM3*/+;*OK107-GAL4*) (Table 1, compare rows 16 and 23) that lacked the *UAS-unf*^*rsqF *^transgene. All MB lobes were observed in all other control pupae (Table 1, rows 19 to 22). MB lobes appeared thin and less robust in some *unf*^*X1 *^and *unf*^*Z0001 *^rescued animals, suggesting that the rescue was imperfect. Nonetheless, MB axons contributing to each of the five MB lobes could be identified in all rescued animals. An independent rescue line, *UAS-unf*^*rsqC*^, was tested with *OK107-GAL4 *to express *unf *in the MBs of *unf*^*X1*^/*Df2426 *hemizygotes. All MB lobes were observed in six of seven rescued *unf*^*X1*^/*Df2426 *pupae with the *UAS-unf*^*rsqC *^transgene (*unf*^*X1*^,*UAS-GFP/Df2426;UAS-unf*^*rsqC*^/+;*OK107-GAL4*). In the seventh pupa, medial lobes were observed bilaterally, while dorsal lobes were observed only in one hemisphere (Table 1, row 17). MB lobes were not detected in any *unf*^*X1*^/*Df2426 *control siblings that lacked the *UAS-unf*^*rsqC *^transgene (*unf*^*X1*^,*UAS-GFP/Df2426;TM3*/+;*OK107-GAL4*; Table 1, row 24). To confirm that the rescue depended upon the expression of an *unf *open reading frame, we tested the ability of a *UAS-lacZ *transgene to rescue *unf*^*X1*^/*Df2426 *hemizygotes. As expected, MB lobes were not observed in *unf*^*X1*^/*Df2426 *pupae containing the *UAS-lacZ *transgene (*unf*^*X1*^,*UAS-GFP/Df2426;UAS-lacZ/+;OK107-GAL4*; Table 1, row 25). These data demonstrate that the axonal defects observed in *unf*^*X1 *^mutants are due to the lack of UNF function.

It is interesting to note that the medially projecting axons of any genotype carrying the *UAS-unf*^*rsqF *^and *OK107-GAL4 *transgenes failed to stop appropriately, extending axons past the midline. Midline crossing was observed in mutants carrying *UAS-unf*^*rsqF *^and *OK107-GAL4 *(Figure 1E) as well as controls carrying these two transgenes. Midline crossing was observed approximately 50% of the time: *unf*^*X1*^,*UAS-GFP/Df2426;UAS-unf*^*rsqF*^/+;*OK107-GAL4 *rescue animals (55%, *n *= 11), *unf*^*X1*^,*UAS-GFP/CyO;UAS-unf*^*rsqF*^/+;*OK107-GAL4 *control siblings (44%, *n *= 9), and *UAS-GFP*/+;*UAS-unf*^*rsqF*^/+;*OK107-GAL4 *controls (60%, *n *= 5). This observation suggests that MB axons may be sensitive to levels of UNF expression.

### *unf*^*X1*^/*unf*^*Z0001 *^compound heterozygotes exhibit a range of mushroom body phenotypes

**Figure 3:**
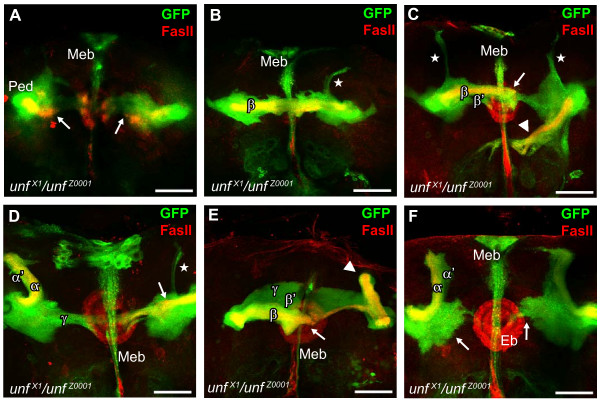
***unf*^*X1*^/*unf*^*Z0001 *^compound heterozygotes display a range of aberrant mushroom body phenotypes, suggesting that *unf*^*X1 *^and *unf*^*Z0001 *^alleles interact**. *OK107-GAL4 *was used to drive expression of the *UAS-mCD8GFP *transgene in all MB neurons and their axons (green), and anti-Fas II (red) to label the α and β axons. **(A) **In this *unf*^*X1*^/*unf*^*Z0001 *^compound heterozygote all MB axons stall (arrows), similar to the *unf*^*X1 *^mutant phenotype. **(B) **In this *unf*^*X1*^/*unf*^*Z0001 *^heterozygote only medial lobes are present, similar to the *unf*^*Z0001 *^mutant phenotype; a thin fascicle of α' axons is present in the right hemisphere (star). **(C) **In this *unf*^*X1*^/*unf*^*Z0001 *^heterozygote, left hemisphere β' and β axons extend medially beyond the midline (arrow), whereas γ axons appear to stall; right hemisphere β and β' axons misproject ventrally (arrowhead) and γ axons are highly disorganized; only a few α' dorsal axon projections are present in either hemisphere (stars). **(D) **In this *unf*^*X1*^/*unf*^*Z0001 *^heterozygote α and α' dorsal axon projections are present in the left hemisphere, whereas only a thin fascicle of α' axons is present in the right hemisphere (star); β axons are present in the right hemisphere and appear to stall (arrow), whereas they are completely absent in the left hemisphere. **(E) **In this *unf*^*X1*^/*unf*^*Z0001 *^heterozygote β', β, and γ axons project medially and cross the midline (arrow), but dorsal axons are missing in the left hemisphere; in the right hemisphere γ axons project medially and α and α' axons project dorsally but appear to stall (arrowhead). **(F) **In this *unf*^*X1*^/*unf*^*Z0001 *^heterozygote, α and α' dorsal axon projections are present in the left hemisphere, whereas only α' dorsal axons are present in the right hemisphere; medial axon projections are disorganized and stall before reaching the midline in both right and left hemispheres (arrows). Eb, ellipsoid body; Meb, median bundle; Ped, peduncle. Scale bars = 25 μm.

Phenotypic analysis of *unf*^*X1*^/*unf*^*Z0001 *^compound heterozygotes (*unf*^*X1*^,*UAS-GFP/unf*^*Z0001*^;;*OK107-GAL4*) revealed a range of aberrant MB phenotypes. Thirteen percent of *unf*^*X1*^/*unf*^*Z0001 *^compound heterozygotes lacked all MB lobes (Figure 3A), similar to the *unf*^*X1 *^mutant phenotype. Forty-seven percent of the *unf*^*X1*^/*unf*^*Z0001 *^compound heterozygotes developed only medial lobes and were missing dorsal lobes (Figure 3B, C), similar to the *unf*^*Z0001 *^mutant phenotype. These *unf*^*Xl*^/*unf*^*Z0001 *^compound heterozygotes occasionally displayed a thin fascicle of dorsally projecting α' axons (Figure 3B, C). Interestingly, 40% of *unf*^*X1*^/*unf*^*Z0001 *^compound heterozygotes exhibited asymmetrical phenotypes in which a dorsal and/or medial lobe were present in one hemisphere but missing in the other (Table 1, row 14). In some cases, medial axons misprojected or extended past the midline (Figure 3B, C, E). γ neurons were also variably affected in *unf*^*X1*^/*unf*^*Z0001 *^compound heterozygotes and often appeared defasciculated, and stalled at various points along their medial trajectory (Figure 3C, D, F; Additional files 6, 7 and 8). The novel phenotypes of *unf*^*X1*^/*unf*^*Z0001 *^compound heterozygotes that are different from either *unf*^X1^/*Df2426 *or *unf*^*Z0001*^/*Df2426 *hemizygotes demonstrate that the *unf*^*X1 *^and *unf*^*Z0001 *^alleles interact.

### *unf *is required for larval-specific γ dorsal collaterals and the re-extension of γ axons during metamorphosis

The axon stalling phenotypes of *unf*^*X1*^/*Df2426 *hemizygotes suggested that *unf *is required in all MB neurons for axons to extend in any direction beyond the heel region of the MB. This region is the branching point for dorsal collateral projections from medially projecting axons. The MB axons of adult *unf*^*X1*^/*Df2426 *hemizygotes may have all stalled during the initial phase of their outgrowth, either during larval or pupal development. Alternatively, it is possible that MB neurons may initially project axons medially and dorsally, but these axons may not be maintained into the adult. To determine whether *unf *is required for the initial projection patterns of all MB neurons, the MBs of experimental and control animals were analyzed at various larval and pupal stages.

**Figure 4:**
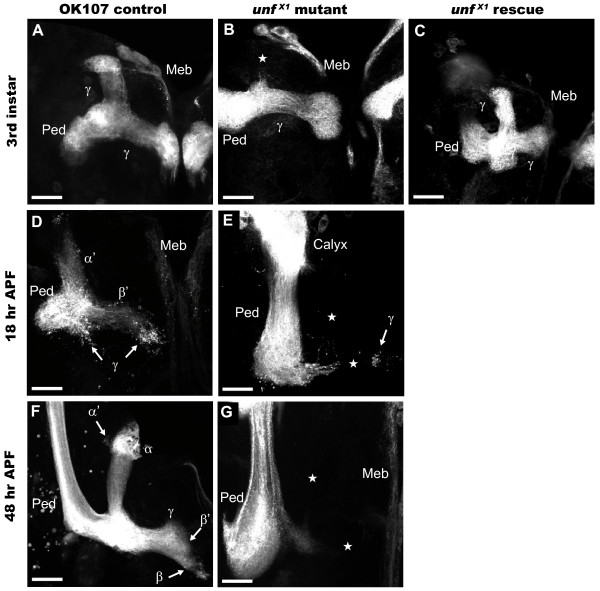
***unf *impacts all mushroom body neurons early during development. *OK107-GAL4 *was used to drive expression of the *UAS-mCD8GFP *transgene in all MB neurons and their axons (white)**. **(A) **In *UAS-mCD8GFP;;OK107-GAL4 *late third instar larvae controls, γ neurons project larval-specific axons medially and dorsally. **(B) **In *unf*^*X1 *^mutant late third instar larvae, γ neurons project medially only; dorsal axons are missing (star). **(C) **Medial and dorsal γ axons are present in *unf*^*X1 *^rescue third instar larvae. **(D) **In *UAS-mCD8GFP;;OK107-GAL4 *control pupae at 18 hours APF, γ axons have been pruned back to the peduncle and only axon fragments are visible; some β' and α' axons project medially and dorsally, respectively. **(E) **In *unf*^*X1 *^mutant pupae at 18 hours APF, γ axons have been pruned with some fragments visible (arrow); β' and α' axons are completely missing (stars). **(F) **All MB lobes are visible in *UAS-mCD8GFP;;OK107-GAL4 *control pupae at 48 hours APF. **(G) **All MB lobes are absent in *unf*^*X1 *^mutant pupae at 48 hours APF (stars). Scale bars = 10 μm.

During late embryonic development, γ neurons normally begin to extend axons both medially and dorsally. These medial and dorsal projections persist throughout larval development. By 18 hours APF, γ axons have been pruned to the branching point and they subsequently re-extend medially only [3-5]. *unf*^*X1*^/*Df2426 *(*unf*^*X1*^,*UAS-GFP/Df2426;;OK107-GAL4*) early-, mid-, and late-third instar larvae displayed only medial axons (*n *= 17), whereas control (*UAS-GFP;;OK107-GAL4*) third instar larvae displayed normal bifurcated larval-specific γ projection patterns (*n *= 5) (compare Figure 4A and Figure 4B). Examination of *unf*^*X1*^/*Df2426 *hemizygotes (*n *= 4) and control (*n *= 6) pupae at 18 hours APF revealed that both medial and dorsal γ axons had been pruned (compare Figure 4D and Figure 4E). At 48 hours APF, MB lobes were not observed in *unf*^*X1*^/*Df2426 *hemizygotes (*n *= 3), whereas control pupae exhibited γ axons that had re-extended medially, forming the adult γ lobe (*n *= 5) (compare Figure 4F and Figure 4G). These data suggest that γ neurons extend axons medially but not dorsally in *unf*^*X1*^/*Df2426 *larvae and that they undergo pruning like wild-type axons at approximately 16 hours APF but that they fail to re-extend during pupal development.

Expression of the *UAS-unf*^*rsqF *^transgene in the MBs in *unf*^*X1*^/*Df2426 *hemizygotes (*unf*^*X1*^,*UAS-GFP/Df2426;UAS-unf*^*rsqF*^/+;*OK107-GAL4*) rescued the dorsal collaterals of the larval γ neurons in five of six third instar larvae (Figure 4C; Table 1, row 15) and supported the re-extension of γ medial axon projections in pupae (Figure 1E, 1F). These data confirm that the *unf *function in MBs is necessary for the formation of dorsal collaterals in γ neurons during larval development.

### *unf *is required during the early development of α'/β' and α/β mushroom body neurons

The α'/β' neurons develop medial and dorsal projections between mid-third instar and puparium formation. The α/β axons develop during early pupal stages [3-5]. α'/β' medial or dorsal projections were never observed in *unf*^*X1*^/*Df2426 *(*unf*^*X1*^,*UAS-GFP/Df2426;;OK107-GAL4*) late-third instar larvae (*n *= 5) or pupae at 18 hours APF (*n *= 6; Figure 4E). Similarly, α'/β' and α/β projections were never observed in *unf*^*X1*^/*Df2426 *pupae at 48 hours APF (*n *= 3; Figure 4G). MB axon projections were normal in control (*UAS-GFP;;OK107-GAL4*) late-third instar larvae (*n *= 5), pupae at 18 hours APF (*n *= 6; Figure 4D), and pupae at 48 hours APF (*n *= 5; Figure 4F). These data indicate that *unf *is necessary for the differentiation of all α'/β' and α/β MB neurons early during their development.

### *201Y-GAL4 *and *c739-GAL4 *driven GFP expression in the mushroom bodies is *unf*-dependent

**Figure 5:**
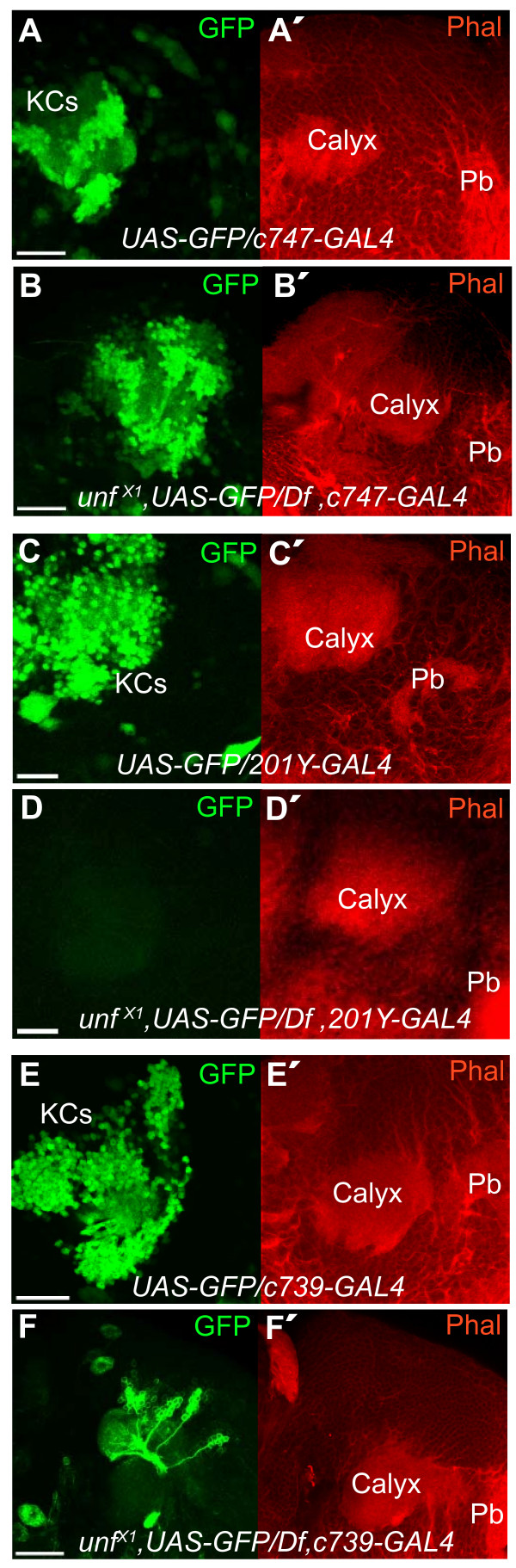
***201Y-GAL4 *and *c739-GAL4 *driven GFP expression is *unf*-dependent**. The *UAS-mCD8GFP *reporter was used with three enhancer trap constructs to visualize MB neurons (Kenyon cells (KCs), green). Fluorescently labeled phalloidin (red) was used to visualize actin-rich structures, including the dendritic fields (calyces) of the MBs and the protocerebral bridge (Pb) that lies in the same plane. **(A, B) **The *c747-GAL4 *transgene drives the expression of GFP in MB neurons in *UAS-mCD8GFP/c747-GAL4 *controls and in *unf*^*X1*^,*UAS-mCD8GFP/Df2426,c747-GAL4 *mutants. **(C, D) **The *201Y-GAL4 *transgene drives the expression of GFP in a subset of the MB neurons in *UAS-mCD8GFP/201Y-GAL4 *controls (C), but *unf*^*X1*^,*UAS-mCD8GFP/Df2426,201Y-GAL4 *mutants do not express *201Y-GAL4*-driven GFP in MB neurons (D). **(E, F) ***UAS-mCD8GFP/c739-GAL4 *controls express GFP in MB neurons (E). GFP expression is greatly reduced in *unf*^*X1*^,*UAS-mCD8GFP/Df2426,c739-GAL4 *mutants (F). The calyx and Pb are positively labeled with phalloidin in all mutants and controls. Scale bars = 10 μm.

To independently confirm that the axon stalling phenotypes of *unf*^*X1*^/*Df2426 *hemizygotes were not an artifact associated with the *OK107-GAL4 *transgene (Figure 1), we examined *UAS-GFP *expression in the MBs of *unf*^*X1*^/*Df2426 *hemizygotes using three other GAL4 drivers known to express in all or subsets of MB neurons, *201Y-GAL4*, *c739-GAL4*, and *c747-GAL4 *[7,40]. In each case, adult brains were counterstained with fluorescently labeled phalloidin to visualize actin-rich structures, including the extensive dendritic arbors of MB neurons that fill the calyces. The *c747-GAL4 *line showed GFP expression in MB neurons in control and *unf*^*X1*^/*Df2426 *MB neurons (Figure 5A, 5B). The *unf*^*X1*^/*Df2426 *hemizygotes (*unf*^*X1*^,*UAS-GFP/Df2426,c747-GAL4*) displayed stalled axons and failed to develop any MB lobes, confirming the *unf*^*X1 *^mutant phenotypes described using the *OK107-GAL4 *transgene to drive the reporter transgene in MB neurons. Surprisingly, *unf*^*X1*^/*Df2426 *hemizygotes carrying the *201Y-GAL4 *transgene failed to express GFP in MB neurons (compare Figure 5C and Figure 5D), and GFP expression in *unf*^*X1*^/*Df2426 *hemizygotes carrying the *c739-GAL4 *transgene was greatly diminished (compare Figure 5E and Figure 5F). These observations indicate that *201Y-GAL4 *and *c739-GAL4 *expression is *unf*-dependent. The *201Y-GAL4 *transgene is an insertion in the *TAK1-associated binding protein 2 *(*Tab2*) gene (FlyBase). Inverse PCR revealed that the *c739-GAL4 *transgene is inserted in the second intron of *hormone receptor-like in 39 *(*HR39*; data not shown).

## Discussion

### The *unf*^*X1 *^and *unf*^*Z0001 *^alleles interact showing that both alleles are at least partially functional

*unf *mutants exhibit a range of highly penetrant axon stalling phenotypes affecting all neurons (γ, α'/β' and α/β) of the larval and adult MBs. Similar phenotypes have been observed in *unf *microRNA knockdown animals [41]. *unf*^*X1*^/*Df2426 *hemizygotes and *unf*^*X1*^/*unf*^*X1 *^homozygotes fail to project larval-specific γ dorsal collaterals, fail to re-extend γ axons medially during metamorphosis, and fail to project any medial and dorsal axons of α'/β' and α/β neurons. The γ, α'/β' and α/β axons of *unf*^*Z0001*^/*Df2426 *hemizygotes only project medially, whereas MBs were normal in some *unf*^*Z0001*^/*unf*^*Z0001 *^homozygotes. These data together with previous observations [25] would seem to support the hypothesis that the *unf*^*X1 *^allele is an amorph, a null allele, and that the *unf*^*Z0001 *^allele is a hypomorph, a partial loss of function allele. However, while the *unf*^*Z0001 *^allele behaves as a hypomorph with respect to sterility, it displays dominant properties with respect to wing expansion [25]. Interestingly, the G56R allele of *PNR*, which displays dominant properties [42], is structurally equivalent to the *unf*^*Z0001 *^allele (G120R) [25]. The observation that the *unf*^*X1*^/*unf*^*Z0001 *^compound heterozygotes display unique phenotypes was unexpected and demonstrates that these alleles interact, compelling us to conclude that the *unf*^*X1 *^allele is not a null allele. These data strongly suggest that the *unf*^*X1 *^allele encodes a unique isoform of the UNF protein, UNF^X1^, which is predicted to contain the 110 residue amino-terminal domain and the complete first zinc finger of the DNA-binding domain [25]. These data do not allow us to infer the functional nature of the *unf*^*X1 *^allele or the mechanism of this genetic interaction.

### Asymmetrical phenotypes suggest a role for *unfulfilled *in pioneer axon guidance

The phenotypic variation and asymmetry observed in the MBs of *unf*^*X1*^/*unf*^*Z0001 *^compound heterozygotes supports the hypothesis that pioneer axons are established early during MB development and that the pathfinding of these pioneers is *unf*-dependent. The observation that the β lobe axons in one hemisphere project medially while the β lobe axons in the other hemisphere project ventromedially (Figure 3C, 45° angle down, arrowhead) demonstrates that the projection of the β lobes in these *unf*^*X1*^/*unf*^*Z0001 *^compound heterozygotes is independent of their genotype. In an independent, yet genetically identical animal, *unf*^*X1*^/*unf*^*Z0001 *^α/β MB neurons assume a different fate and fail to project any medially projecting β axons (Figure 3F). Similar observations can be made for all *unf*^*X1*^/*unf*^*Z0001 *^MB lobe axons when these and other samples are examined (Figure 3). The fact that the axons of the later-born α/β neurons consistently stall or misproject whenever the axons of the earlier-born α'/β' neurons stall or misproject suggests that the α'/β' axons may be acting as pioneers for the α/β axons. These data support a model of MB lobe formation in which *unf *is required for MB pioneer axons to navigate to their targets, and that later-born MB neurons project axons that fasciculate along these established axons. We propose that the variable and asymmetric phenotypes observed in *unf*^*X1*^/*unf*^*Z0001 *^compound heterozygotes are due to inappropriate targeting of pioneer axons of the MB or the stalling of pioneer axons prematurely as a result of insufficient *unf *function in α'/β' pioneer axons. Thus, the asymmetric β lobe projection in Figure 3C may be due to asymmetric projections of pioneer axons, while the lack of β lobes in Figure 3F may be due to the stalling of these pioneer axons in the peduncle. These data are supported by an analysis of non-autonomous effects of *Dscam *mutant clones, which suggests that the α'/β' axons may be acting as pioneers for the α/β axons at least some of the time [43]. Our observations that larval γ dorsal axons, and the α' and α dorsal lobes all fail to develop in *unf*^*Z0001*^/*Df2426 *mutants not only supports the hypothesis that the α'/β' axons act as pioneers for the α/β axons, but suggests that the larval γ axons act as pioneers for the α'/β' axons. Discerning the roles and mechanisms of *unf *in MB pioneers during post-embryonic MB development requires further investigation.

### *unf *plays a common role in the early development of all mushroom body neurons

The data presented here demonstrate that *unf *plays a common role in the early development of all three subtypes of MB neurons by regulating axon extension and branching. While we cannot rule out the possibility that single axons, which normally project dorsally, may be misguided and project medially, our analysis is consistent with the hypothesis that *unf *mutant γ, α'β', and α/β neurons fail to project dorsal axon branches. Our observations that *unf *mutant MB neurons express subtype-specific epitopes such as Fas II and Trio suggest that *unf *does not impact MB neuronal subtype identity. Interestingly, Lin *et al*. [41] disagree and conclude that *unf *does regulate MB neuronal subtype identity based on a series of *unf *RNAi knockdown experiments. We argue that until the transcriptional codes that distinguish MB neuronal subtypes are defined, one cannot conclusively determine whether the identity of these neurons has been impacted [44-47]. While we cannot yet place *unf *at any specific position in a transcriptional hierarchy that regulates MB development, our data suggest that *unf *acts earlier than *dac*, *EcR-B1*, and *usp*, since these genes regulate differentiation in specific subsets of MB neurons while *unf *regulates the differentiation of all three MB subtypes.

Based on the homology of UNF with PNR, we anticipate that UNF is likely to act as a dual function transcription factor with the ability to activate the transcription of some target genes, while repressing others [32,33,35,36]. Palanker *et al*. [48] have previously proposed that UNF may function as a transcriptional repressor. We propose that the axon stalling phenotype observed in *unf *mutants is due to the misregulation of target genes. Two potential target genes are *Tab2 *and *HR39 *based on the observation that the expression of the *201Y-GAL4*, an enhancer trap insertion in *Tab2*, and *c747-GAL4*, an enhancer trap insertion in *HR39*, is *unf*-dependent. If the transcriptional regulation of these two enhancer trap transgenes reflects the transcriptional regulation of the genes in which they are inserted, then it follows that *Tab2 *and *HR39 *are expressed in the MBs and that this expression is *unf*-dependent, a hypothesis that remains to be tested.

Axon stalling and branching phenotypes in *unf *mutants suggest that genes encoding axon guidance cues are likely to be regulated by *unf*. Genetic screens have already identified a number of guidance genes that are expressed in the MBs. For example, genes encoding cell adhesion molecules like *volado*, an α-integrin, *Notch*, a transmembrane receptor and transcription factor [1], and *semaphorinla *[49] and *plexinA *[15], which encode a ligand-receptor pair that is largely involved in axonal repulsion, have been identified. Ephrin receptor tyrosine kinase [50] and Dscam [43,51], other well-known guidance cues, are necessary for the proper guidance of MB axons. Misregulation of these or other guidance genes could disrupt the normal balance of attractive and repulsive cues resulting in inappropriate axon pathfinding and stalling.

## Conclusions

These data support the hypothesis that *unf *plays a common role in the early development of all three subtypes of MB neurons, γ, α'/β', and α/β, by regulating axon extension and branching during the initial phases of larval and pupal outgrowth. Expression of a *UAS-unf *transgene in MB neurons of *unf *mutants rescues the *unf *mutant MB phenotypes, demonstrating that the MB defects are due to the lack of *unf*. The phenotypic variation and asymmetry observed in the MBs of *unf*^*X1*^/*unf*^*Z0001 *^compound heterozygotes suggests a role for *unf *in the targeting of pioneer axons.

## Materials and methods

### *Drosophila *stocks

All stocks were raised on standard cornmeal and sugar medium. The *unf*^*X1 *^and *unf*^*Z0001 *^stocks have been characterized previously: the *unf*^*X1 *^allele disrupts the 5' donor splice site of intron 2, whereas the *unf*^*Z0001 *^allele has a missense mutation due to a guanine to adenine transition at base 312 of exon 2 resulting in a glycine to arginine substitution (G120R) [25]. The *Df(2R)ED2426 *(*Df2426*) chromosome carries a deletion of 482,016 bp on the second chromosome that removes 57 genes or annotated genes in their entirety, including *unf *[52]. The *Hr51*^*MB05909 *^(*unf*^*MB05909*^) line contains a GAL4 insertion in intron 1 of the *unf *gene (FlyBase). The *Df(2R)ED2426*, *Hr51*^*MB05909 *^(*unf*^*MB05909*^), *UAS-CD8::GFP;;OK107-GAL4*, *201Y-GAL4*, *c747-GAL4*, and *c739-GAL4 *lines were obtained from Bloomington Stock Center. The *UAS-unf*^*RNAi *^line was obtained from Vienna *Drosophila *RNAi Center (VDRC). All mutant or transgenic stocks were maintained over GFP-marked chromosomes to facilitate genotyping. Animals were reared at 25°C with the exception of *UAS-unf*^*RNAi *^crosses, which were reared at 29°C.

### Transgenic flies and rescue experiment

The transgenic rescue constructs *UAS-unf*^*rsqF *^and *UAS-unf*^*rsqC *^are two independent isolates of the same transgene, which was generated by cloning the *unf *cDNA [25] into the vector pUAST [40]. Transgenic flies were generated by P-element-mediated transformation [53]. A homozygous *w*^*1118 *^stock was used for all P-element-mediated transformations. *Df2426/CyO;UAS-unf*^*rsq*^/*TM3Sb *flies were crossed to *unf*^*X1*^,*UAS-GFP/CyOGFP;;OK107-GAL4 *or *unf*^*Z0001*^,*UAS-GFP/CyOGFP;;OK107-GAL4 *flies to generate rescues and control siblings. *Df2426/CyO;UAS-lacZ/TM3Sb *flies were crossed to *unf*^*X1*^,*UAS-GFP/CyOGFP;;OK107-GAL4 *flies for additional rescue controls. All rescue and control larvae and pupae were genotyped using PCR. The following primers were used to detect the presence of *unf*^*X1*^, *unf*^*Z0001*^, *Df2426*, and *UAS-unf*^*rsq*^. *unf*^*X1*^, 5' CAGCGGCATTGCTACACTC 3' (fx1b1) and 5' GGAAAATTCCCACGTCAAAA 3' (R947) followed by *Xba*I digest; *unf*^*Z001*^, 5' CTGAGCTGGAATCACAGTGC 3' (L150Z1) and 5' GGATTCCGTAGTGCTTTCT 3' (R330Z1); *Df2426*, 5' TCATTTAATTTTAGTGCCGGA 3' (2426A) and 5' CAATCATATCGCTGTCTCACTCA 3' (PRY4); *UAS-unf*^*rsq*^, 5' CAGCGGCATTGCTACACTC 3' (fx1b1) and 5' GATTCCGATGAGCTTTGTCCACCACAC 3' (XR941).

### Immunohistochemistry and microscopy

Third instar larvae and pupae were staged as described [54,55]. The central nervous system of third instar larvae, pupae, and 0- to 2-day adults were collected, fixed in 4% paraformaldehyde, and processed using standard protocols [9]. mAb1D4 [56] (anti-Fas II; 1:10) and mAb9.4A [39] (anti-Trio; 1:4) were obtained from the Developmental Studies Hybridoma Bank (DSHB). The rabbit anti-Fas II (1:2,000) was a gift from Dr Vivian Budnik (University of Massachusetts). Biotinylated anti-mouse and anti-rabbit IgG (1:200) were obtained from Vector Labs (Burlingame, CA USA). Streptavidin Alexa Fluor 488, 546 (1:200), and Alexa Fluor 546 phalloidin (1:40) were obtained from Invitrogen Molecular Probes (Carlsbad, CA USA). Preparations were examined and imaged using an Olympus Fluoview FV-1000 laser scanning confocal system mounted on an Olympus IX-81 inverted microscope. Images were processed using Image J and Adobe Photoshop. Movies of z stacks were processed using QuickTime. During z stack collections for Additional files 1 to 3, the photomultiplier tube was manually adjusted for optimal brightness at different focal planes.

### Statistics

The Fisher exact test was used to determine whether the frequency of defects in experimental animals was significantly different from the frequency of defects in control animals. Relevant genotypes were tested in pair-wise combinations. *P*-values less than 0.01 were considered significant.

## Abbreviations

APF: after puparium formation; *dac*: *dachshund*; *EcR-B1*: *ecdysone receptor B1*; Fas II: Fasciclin II; *fax-1*: *fasciculation of axons defective*; GFP: green fluorescent protein; *HR39*: *hormone receptor 39*; MB: mushroom body; *PNR: photoreceptor specific nuclear receptor*; RNAi: RNA interference; *Tab2*: *Tak1-associated binding protein*; *usp*: *ultraspiracle*; *unf*: *unfulfilled*.

## Competing interests

The authors declare that they have no competing interests.

## Authors' contributions

KEB and CSS were responsible for all technical work. CSS was solely responsible for generating the rescue animals. KEB was largely responsible for phenotypic analyses and writing the manuscript. SR conceived of, directed, and obtained funding for the study, and participated in writing the manuscript. All authors read and approved the final manuscript.

## Supplementary Material

Additional file 1 **Figure S1**. Movie of a z stack of an adult *unf*^*X1*^,*UAS-mCD8GFP/CyO;;OK107-GAL4 *control animal labeled with anti-Fas II (red), in which all MB lobes are properly formed. *OK107-GAL4 *driven GFP expression is visible in the Kenyon cells (green) and all MB lobes. Images depict the right hemisphere at 90× magnification. Click here for file

Additional file 2 **Figure S2**. Movie of a z stack of an adult *unf*^*X1*^,*UAS-mCD8GFP/Df2426;;OK107-GAL4 *mutant labeled with anti-Fas II (red). The Kenyon cells (green) are visible in posterior planes. A rarely observed medial projection is visible in the region of the calyx. Axons travel anteroventrally then spread out, and stall prior to lobe formation. Images depict the left hemisphere at 90× magnification. Click here for file

Additional file 3 **Figure S3**. Movie of a z stack of an adult *unf*^*X1*^,*UAS-mCD8GFP/Df2426;;OK107-GAL4 *mutant labeled with anti-Fas II (red). Axons project anteroventrally in a poorly organized peduncle, spread out, and stall at the end of the peduncle. The median bundle (green) is to the left. Images depict the right hemisphere at 90× magnification. Click here for file

Additional file 4 **Figure S4**. Movie of a z stack of an adult *unf*^*Z0001*^,*UAS-mCD8GFP/Df2426;;OK107-GAL4 *mutant labeled with anti-Fas II (red). Axons travel anteroventrally. γ, β', and β axons projected medially but were disorganized. The γ axons occupy the most superficial plane and can be distinguished from the more posterior β' (green) and Fas II-positive β (yellow) axons. Images depict the right hemisphere at 90× magnification. Click here for file

Additional file 5 **Figure S5**. Movie of a z stack of an adult *unf*^*Z0001*^,*UAS-mCD8GFP/unf*^*Z0001*^;;*OK107-GAL4 *mutant labeled with anti-Fas II (red). Axons travel anteroventrally in a tightly organized peduncle. γ, β', and β axons projected medially. A thin fascicle of axons projects dorsally but stalls. Images depict the left hemisphere at 90× magnification. Click here for file

Additional file 6 **Figure S6**. Movie of z stacks of adult *unf*^*X1*^,*UAS-mCD8GFP/unf*^*Z0001*^;;*OK107-GAL4 *compound heterozygotes. The γ axons occupy the most superficial plane and can be distinguished from the more posterior β' (green) and Fas II-positive β (yellow) medial axons; the Fas II-positive α' dorsal axons (yellow) can be distinguished from the α dorsal axons (green), which do not express Fas II. Figure S6 corresponds to Figure 3C in the text. Images were obtained at a magnification of 60×. Click here for file

Additional file 7 **Figure S7**. Movie of z stacks of adult *unf*^*X1*^,*UAS-mCD8GFP/unf*^*Z0001*^;;*OK107-GAL4 *compound heterozygotes. The γ axons occupy the most superficial plane and can be distinguished from the more posterior β' (green) and Fas II-positive β (yellow) medial axons; the Fas II-positive α' dorsal axons (yellow) can be distinguished from the α dorsal axons (green), which do not express Fas II. Figure S7 corresponds to Figure 3D. Images were obtained at a magnification of 60×. Click here for file

Additional file 8 **Figure S8**. Movie of z stacks of adult *unf*^*X1*^,*UAS-mCD8GFP/unf*^*Z0001*^;;*OK107-GAL4 *compound heterozygotes. The γ axons occupy the most superficial plane and can be distinguished from the more posterior β' (green) and Fas II-positive β (yellow) medial axons; the Fas II-positive α' dorsal axons (yellow) can be distinguished from the α dorsal axons (green), which do not express Fas II. Figure S8 corresponds to Figure 3F. Images were obtained at a magnification of 60×. Click here for file
